# Stressful life events, social health issues and low birthweight in an Australian population-based birth cohort: challenges and opportunities in antenatal care

**DOI:** 10.1186/1471-2458-11-196

**Published:** 2011-03-30

**Authors:** Stephanie J Brown, Jane S Yelland, Georgina A Sutherland, Peter A Baghurst, Jeffrey S Robinson

**Affiliations:** 1 Healthy Mothers Healthy Families Research Group, Murdoch Childrens Research Institute, Melbourne, Australia; 2 Department of General Practice and School of Population Health, University of Melbourne, Melbourne, Australia; 3 Women's and Children's Hospital, University of Adelaide, Adelaide, Australia

## Abstract

**Background:**

Investment in strategies to promote 'a healthy start to life' has been identified as having the greatest potential to reduce health inequalities across the life course. The aim of this study was to examine social determinants of low birthweight in an Australian population-based birth cohort and consider implications for health policy and health care systems.

**Methods:**

Population-based survey distributed by hospitals and home birth practitioners to >8000 women six months after childbirth in two states of Australia. Participants were women who gave birth to a liveborn infant in Victoria and South Australia in September/October 2007. Main outcome measures included stressful life events and social health issues, perceived discrimination in health care settings, infant birthweight.

**Results:**

4,366/8468 (52%) of eligible women returned completed surveys. Two-thirds (2912/4352) reported one or more stressful life events or social health issues during pregnancy. Women reporting three or more social health issues (18%, 768/4352) were significantly more likely to have a low birthweight infant (< 2500 grams) after controlling for smoking and other socio-demographic covariates (Adj OR = 1.77, 95% CI 1.1-2.8). Mothers born overseas in non-English speaking countries also had a higher risk of having a low birthweight infant (Adj OR = 1.85, 95% CI 1.2-2.9). Women reporting three or more stressful life events/social health issues were more likely to attend antenatal care later in pregnancy (OR = 2.06, 95% CI 1.3-3.1), to have fewer antenatal visits (OR = 2.17, 95% CI 1.4-3.4) and to experience discrimination in health care settings (OR = 2.69, 95% CI 2.2-3.3).

**Conclusions:**

There is a window of opportunity in antenatal care to implement targeted preventive interventions addressing potentially modifiable risk factors for poor maternal and infant outcomes. Developing the evidence base and infrastructure necessary in order for antenatal services to respond effectively to the social circumstances of women's lives is long overdue.

## Background

Several recent reports, including the *Fair Society, Healthy Lives *report (The Marmot Review) and World Health Organisation's *Commission on Social Determinants of Health *(CSDH) *Closing the Gap in a Generation*, have identified investment in strategies to promote 'a healthy start to life' as having the greatest potential to reduce health inequalities across the life course [1-3]. 'Giving every child the best start in life' was nominated by the Marmot Review as the number one priority for implementation [1]. Central to this recommendation is the accumulating evidence to show that brain development is highly sensitive to external influences *in utero *and in early childhood, with potential life long effects [4,5]. Infants that are born preterm, small for gestational age and/or with a low birthweight have well documented increased health risks in childhood, with more recent evidence supporting effects continuing into later life [6]. This research provides a powerful argument for supporting early intervention to improve maternal health in pregnancy and pre-conception as part of a comprehensive strategy to improve maternal, newborn and child health.

The major contribution of the *CSDH Report *and the *Marmot Review *is to situate this strategy within the broader context of health equity and the social determinants of health [1,2]. There is mounting evidence in both a developing and a developed country context, demonstrating higher prevalence of low birthweight and small for gestational age infants associated with individual and area-level measures of social adversity and deprivation [7-14]. Worse outcomes have been reported for 'blue collar workers' compared with 'white collar workers' [10,12], lone parents [8,11], low income households [11], and mothers living in deprived neighbourhoods [11,13,14]. A small number of studies have reported a positive association between low birthweight and stressful life events during or immediately preceding pregnancy [15,16]. Intimate partner violence, trait anxiety in the second and third trimester, and maternal distress in pregnancy also show associations with low birthweight [19-23]. Two potential pathways have been identified to explain these findings. The first posits that psychosocial factors such as stress or lack of social support impact on birth outcomes via neuroendocrine, immune or vascular mechanisms [14,16,17]. The second, more well-established pathway is via health behaviours which are known to be associated with increased risk such as smoking, maternal nutrition, infection and substance use [24].

The overall aim of this paper is to examine social determinants of low birthweight in an Australian population-based cohort and consider implications for health policy and health systems. The paper draws on data collected in an Australian population based survey of 4,366 women who gave birth in South Australia and Victoria to examine (i) the prevalence of stressful life events and social health issues in the 12 months before the birth, and (ii) women's experience of discrimination in health care settings as factors which may potentially contribute to poor infant health outcomes. To our knowledge, discrimination in maternal health care has not previously been investigated as a factor which may contribute to poor maternal and/or child health outcomes. Post hoc analyses are reported examining associations with attendance at antenatal care and with infant birthweight.

## Method

### Sample

The 2008 Healthy Mothers Healthy Families (HMHF) Survey questionnaire was mailed to women giving birth in two Australian states (Victoria and South Australia) in September/October 2007 at five to six months postpartum, excluding those who had a stillbirth, or whose baby was known to have died. Public and private maternity hospitals and homebirth practitioners in both states distributed questionnaires to women who gave birth under their care in the study period. All hospitals with births in the study period (n = 110) agreed to participate, however one small public hospital with approximately 90 confinements in the study period subsequently withdrew. Together South Australia and Victoria accounted for greater than 31% of the 289,496 births in Australia in 2007 [25]. The inclusion of two states provided for greater diversity in the sampled population compared with previous Victorian surveys [26-28]. Compared with Victoria, South Australia has a higher proportion of births to women living in outer regional (11.8% versus 4.2%) and remote/very remote areas (3.9% versus 0%), a higher proportion of Aboriginal mothers (3.0% versus 1.0%) and women giving birth under 20 years (4.6% versus 2.6%), while Victoria has a higher proportion of births to women born overseas (26.6% versus 16.3%) [25].

### Procedure

The primary aim of the study was to assess women's views and experiences of care received during pregnancy, birth and the postnatal period in representative samples of women giving birth in South Australia and Victoria in 2007. Questionnaires, together with a covering letter inviting women to take part, an explanation of the study in six community languages (Arabic, Vietnamese, Cantonese, Mandarin, Somali and Turkish) and a reply paid envelope for returning the questionnaire free of charge, were posted to women at six months postpartum. Two reminders were sent at two-week intervals with the second including a repeat copy of the questionnaire. The timing of the survey at five to six months postpartum matches previous Victorian surveys [26-28] and was decided taking into account the literature suggesting a 'halo' effect in relation to reporting of women's experiences of maternity care in the period immediately after childbirth [27] and the inclusion of questions on postpartum maternal health outcomes (e.g. maternal depression, urinary incontinence). A copy of the questionnaire is available on the study website: http://www.mcri.edu.au/HMHFSurvey. The study period - four weeks in September 2007 in Victoria, and eight weeks in September/October 2007 in South Australia - was chosen to ensure sub-groups of interest (e.g. women attending six main models of care, women <25 years, single women, and women whose infant was <2,500 grams at birth) were large enough for meaningful statistical comparisons.

### Questionnaire

Information was collected on women's views and experiences of care, health service use, maternal socio-demographic characteristics, reproductive history, and maternal and infant health outcomes, including infant birthweight. The questionnaire also included questions on maternal smoking in pregnancy, maternal pre-pregnant weight and height, and stressful life events and social health issues in the 12 months before the index birth. Measures of socio-economic status included: pre-tax household income adjusted for household size and composition, possession of a current health care concession card and health insurance status (private cover/Medicare only). Women were classified as being at higher risk of complications in pregnancy if they had a previous low birthweight infant, preterm birth, or stillbirth, or if they had any substantial medical or obstetric risk factors in the current pregnancy (e.g. multiple pregnancy, gestational diabetes). Maternal pre-pregnant Body Mass Index (BMI) was calculated as the ratio of body weight in kilograms divided by the square of height in metres. Smoking status was assessed using a multiple response question enabling women to indicate if they had never smoked, quit smoking before their pregnancy, quit since the beginning of pregnancy, cut down cigarette smoking since finding out they were pregnant, continued to smoke at about the same level or increased their smoking since the beginning of pregnancy [29]. Women who said they had quit before pregnancy or never smoked were categorised as non-smokers. Women who said they quit when they found out they were pregnant were categorised as smoking in early pregnancy; women who cut down, increased or continued to smoke about the same number of cigarettes as before their pregnancy were categorised as continuing to smoke throughout their pregnancy.

Assessment of stressful life events and social health issues drew on items included in the PRAMS study [30] with additional items incorporated based on results of a pilot survey and consultations with Aboriginal community organisations and communities in the two states. Women were asked "Did any of the following things happen to you in the 12 months before your new baby was born?" and invited to tick 'yes' or 'no' to 23 items including major life events, such as separation and divorce, moving house, losing your job, death of a close family member or friend and social health issues, such as having a lot of bills you couldn't pay, not having enough money to buy food, legal troubles or being involved in a court case, serious family conflict or being homeless. A complete list is provided in an additional file (see Additional File 1). Perceived discrimination was assessed using five questions (see Additional File 1) adapted from the Measure of Indigenous Racism Experience [31] to elicit information about women's experience of being discriminated against by health professionals in the perinatal period. The questions were designed to capture the concept of being 'treated unfairly', that is being treated as inferior, with less respect or courtesy, or receiving poorer care than other people. Women were also asked if they had been insulted, stereotyped, talked down to or ignored. Although based on a measure originally designed to assess racial discrimination, the questions were framed to illicit experiences of perceived discrimination in a variety of other contexts, for example, in relation to maternal age, education and other social characteristics.

The study was approved by the Royal Children's Hospital Human Research Ethics Committee (HREC), Victorian Department of Human Services HREC, South Australian Department of Health HREC and a number of other university and hospital based HRECs.

### Analysis

Data on the social and obstetric characteristics of study participants were compared with routinely collected Victorian and South Australian perinatal data for all women who gave birth in the study period in order to assess the representativeness of the sample. Reporting of stressful life events and social health issues was categorised according to whether women reported no social health issues, one or two issues, or three or more issues. Data on discrimination were dichotomised to distinguish women who reported any experience of discrimination from those who reported never experiencing any of the kinds of discrimination we asked about. Data were analysed using STATA version 11.0 [32] and involved the calculation of unadjusted and adjusted odds ratios and 95% confidence intervals. Multivariable logistic regression was used to assess the relationship between number of stressful life events and social health issues as the exposure of main interest (categorised as none, one to two issues, three or more issues) and low birthweight as the primary outcome variable. Four models are reported adjusting for: maternal smoking, social characteristics, number of antenatal visits and medical risk.

## Results

Questionnaires were mailed to 8,597 women. The adjusted response fraction excluding a small number of questionnaires (n = 129) 'returned to sender', duplicate responses and women who gave birth outside the study period was 52% (4366/8468).

### Social characteristics and risk factors

Table 1 shows the social and obstetric characteristics of participants compared with all Victorian and South Australian women who gave birth in the study period according to routinely collected perinatal data for both states. The mean age of women in the sample was 31 years (range 16 to 46 years). Comparisons with routinely collected data showed that women taking part in the survey were largely representative in terms of key obstetric characteristics including parity, method of birth, and infant birthweight, but included fewer women born overseas of non-English speaking background, Aboriginal and Torres Strait Islander women, single women and women under 25 years. Eighteen percent of women reported smoking in pregnancy, including women who said that they quit or cut down smoking while pregnant. One third of participants who provided data on height and weight prior to pregnancy were overweight (25.0-29.9) or obese (≥30). Two-thirds of women reported one or more stressful life events or social health issues during pregnancy, and 17% reported three or more issues.

**Table 1:** Social and obstetric characteristics of participants in the 2008 HMHF Survey and all women who gave birth in Victoria and South Australia during the study period*

	HMHF Survey participants		All women who gave birth in study period	
	**No**.	**%**	**No**.	**%**

**Maternal age**				
≤24 years	416	9.9	1385	16
25-29 years	1053	25	2301	26.7
30-34 years	1604	38.1	2928	33.8
≥35 years	1138	27	2035	23.5

**Relationship status**				
Married or living with partner	4201	95.5	7573	87.6
Single	144	3.3	977	11.3
Divorced, separated or widowed	55	1.2	88	1.1

**Equivalised household income (before tax) per annum (AUD)**				
≤ $20,000	938	24.2	Not collected	
$21,000-$40,000	1959	50.5		
≥$41,001	979	25.3		

**Health care concession card**				
Yes	949	21.9	Not collected	
No	3394	78.2		

**Educational attainment (secondary)**				
Completed secondary school	3429	79.3	Not collected	
Did not complete secondary school	894	20.7		

**Educational attainment (tertiary)**				
Completed tertiary education	2582	60	Not collected	
Did not complete tertiary education	1722	40		

**Mother's Country of birth**				
Australia	3521	81.5	6605	76.3
Overseas - English speaking	272	6.3	478	5.6
Overseas - Non-English speaking	526	12.2	1567	18.1

**Risk of medical complications in pregnancy**				
Higher risk	1712	39.2	Not available in both states	
Lower risk	2654	60.8		

**Smoking in pregnancy**				
Yes	752	17.6	Not available in both states	
No	3516	82.4		

**Maternal pre-pregnant BMI**				
Underweight (< 18.5)	182	4.5	Not available in both states	
Normal weight (18.5-24.9)	2400	58.9		
Overweight (25.0-29.9)	909	22.3		
Obese (≥30)	583	14.3		

**Social health issues/stressful life events (12 months before birth)**				
None	1440	33.1	Not available in both states	
One to two issues	2144	49.3		
≥3 issues	768	17.7		

**Parity**				
First baby	1941	44.5	3609	41.6
Second or subsequent baby	2425	55.5	5060	58.4

**Infant birthweight**				
< 2500 g	179	4.1	565	6.5
2500 g-3999 g	3413	78.2	7183	81.9
≥4000 g	560	13.5	1019	11.6

* Data on all women who gave birth in the study period obtained from the Victorian Perinatal Data Collection Unit and South Australian Pregnancy Outcome Unit; totals vary because of missing values

### Stressful life events and social health issues

Table 2 summarises data on associations between maternal social characteristics and stressful life events/social health issues experienced in the 12 months before the birth. Maternal characteristics that were associated with substantially raised odds of reporting three or more stressful life events or social health issues were: being under 25 years; single, divorced or widowed; having a below average equivalised income; not completing year 12; smoking at any stage of pregnancy; being underweight (BMI <18.5); being at higher risk of obstetric complications, and being of Aboriginal and/or Torres Strait Islander origin.

**Table 2:** Relationship between stressful life events/social health issues, perceived discrimination and social and obstetric characteristics

	No social health issues No. (%)	One to two social health issues No. (%)	Three or more social health issues No. (%)	Odds Ratio (95% CI)*	Never experienced discrimination No. (%)	Experienced discrimination No. (%)	Odds Ratio (95% CI)**
**Maternal age**							

≤24 years	84 (20.3)	166 (40.2)	163 (39.5)	**4.48** **(3.4-5.9)**	250 (60.7)	162 (39.3)	**2.00** **(1.6-2.5)**
≥25 years	1315 (34.7)	1900 (50.2)	570 (15.1)	**1.00 ref**	2853 (75.6)	923 (24.4)	**1.00 ref**

**Adjusted household income (AUD)**							
< $20,000	239 (25.6)	400 (42.9)	296 (31.7)	**2.95** **(2.4-3.7)**	619 (66.5)	312 (33.5)	**1.50** **(1.3-1.8)**
$20,000- $40,000	684 (35.0)	983 (50.3)	287 (14.7)	**1.00 ref**	1463 (74.9)	490 (25.1)	**1.00 ref**
> $40,000	364 (37.3)	524 (53.7)	88 (9.0)	**0.58** **(0.4-0.8)**	764 (78.0)	215 (22.0)	**0.84** **(0.7-1.0)**

**Educational attainment**							
Completed Yr 12	1152 (33.7)	1739 (50.9)	529 (15.5)	**1.00 ref**	2556 (74.9)	856 (25.1)	**1.00 ref**
Did not complete Yr 12	272 (30.6)	390 (43.8)	228 (25.6)	**1.83** **(1.5-2.3)**	617 (69.6)	270 (30.4)	**1.31** **(1.1-1.5)**

**Indigenous status**							
Not Aboriginal and/or Torres Strait Islander	1328 (33.2)	1999 (50.1)	713 (16.7)	**100 ref**	2968 (73.6)	1067 (26.4)	**1.00 ref**
Aboriginal and/or Torres Strait Islander	8 (25.8)	11 (35.5)	12 (38.7)	**2.79** **(1.1-6.9)**	21 (67.7)	10 (32.3)	**1.32** **(0.6-2.8)**

**Maternal country of birth**							
Australia	1165 (33.2)	1716 (48.8)	633 (18.0)	**1.00 ref**	2604 (74.2)	906 (25.8)	**1.00 ref**
Overseas - English speaking	82 (30.5)	149 (55.4)	38 (14.1)	**0.85** **(0.5-1.3)**	195 (72.0)	76 (28.0)	**1.12** **(0.8-1.5)**
Overseas - Non English speaking	178 (34.0)	258 (49.3)	87 (16.6)	**0.90** **(0.7-1.2)**	381 (73.1)	140 (26.9)	**1.06** **(0.8-1.3)**

**Relationship status**							
Married	1181 (35.7)	1696 (51.2)	437 (12.2)	**1.00 ref**	2511 (76.3)	779 (23.7)	**1.00 ref**
Living with partner	236 (28.3)	379 (46.8)	219 (26.3)	**2.51** **(2.0-3.1)**	555 (66.9)	274 (33.1)	**1.59** **(1.3-1.9)**
Single/divorce/separated	20 (10.4)	61 (38.5)	111 (57.8)	**15.0** **(9.2-24.4)**	119 (62.6)	71 (37.4)	**1.92** **(1.4-2.6)**

**Maternal pre-pregnant BMI**							
Underweight (< 18.5)	46 (25.3)	91 (50.0)	45 (24.7)	**2.08** **(1.4-3.2)**	121 (68.0)	57 (32.0)	**1.44** **(1.0-2.0)**
Normal weight (18.5-24.9)	814 (34.0)	1197 (50.0)	382 (16.0)	**1.00 ref**	1802 (75.5)	586 (24.5)	**1.00 ref**
Overweight (25-29.9)	308 (34.0)	435 (48.1)	162 (17.9)	**1.12** **(0.9-1.4)**	669 (74.0)	235 (26.0)	**1.08** **(0.9-1.3)**
Obese (≥30)	184 (31.6)	284 (48.7)	115 (19.7)	**1.33** **(1.0-1.7)**	412 (71.0)	168 (29.0)	**1.25** **(1.0-1.5)**

**Smoking during pregnancy**							
Non-smoker	1232 (35.1)	1760 (50.2)	514 (14.7)	**1.00 ref**	2623 (75.2)	864 (24.8)	**1.00 ref**
Quit in pregnancy	95 (27.1)	169 (48.2)	87 (24.8)	**2.20** **(1.6-3.0)**	248 (71.5)	99 (28.5)	**1.21** **(0.9-1.5)**
Smoking throughout pregnancy	86 (21.5)	162 (40.5)	152 (38.0)	**4.24** **(3.2-5.6)**	245 (62.0)	150 (38.0)	**1.86** **(1.5-2.3)**

**Medical risk**							
Lower risk	960 (36.3)	1295 (48.9)	392 (14.8)	**1.00 ref**	2008 (76.3)	624 (23.7)	**1.00ref**
Higher risk	480 (28.2)	849 (49.8)	376 (22.1)	**1.92** **(1.6-2.3)**	1184 (70.0)	508 (30.0)	**1.38** **(1.2-1.6)**

**Gestation**							
≥37 weeks	1333 (33.7)	1939 (49.0)	686 (17.33)	**1.00 ref**	2913 (74.1)	1020 (25.9)	**1.00 ref**
< 37 weeks	79 (28.3)	146 (52.3)	54 (19.4)	**1.32** **(0.9-1.9)**	198 (71.2)	80 (28.8)	**1.15** **(0.9-1.5)**

**Infant birthweight**							
≥2500 grams	1332 (33.6)	1954 (49.3)	676 (17.1)	**1.00 ref**	2903 (73.8)	1032 (26.2)	**1.00 ref**
< 2500 grams	44 (24.7)	88 (49.4)	46 (25.8)	**2.06** **(1.3-3.1)**	121 (68.0)	57 (32.0)	**1.32** **(1.0-1.8)**

* Odds ratios in this column refer to comparisons between women who experienced ≥2 stressful life events with those who experienced no events

** Odds ratios in the last column refer to comparisons between women who experienced discrimination with those reporting no discrimination

### Experiences of discrimination in health care settings

Women were significantly more likely to report experiencing discrimination if they were: under 25 years, had a low income, had not completed year 12, were not married, had a very high (≥35) or a very low BMI (< 18.5), quit smoking in pregnancy or smoked throughout pregnancy and if they were at higher risk of medical complications. Women who reported three or more stressful life events/social health issues were significantly more likely to report discrimination (314/760, 41.3% versus 296/1429, 20.7%, OR = 2.69, 95% CI 2.2-3.3). Women who reported one or two stressful life events/social health issues also had significantly raised odds of experiencing discrimination (518/2123, 24.6% versus 20.7%, OR = 1.2, 95% CI 1.1-1.4) suggesting a 'dose response'.

### Patterns of health service use

Despite the existence of universal health cover via Medicare, uptake of antenatal care varied in relation to maternal social characteristics and the number of stressful life events or social health issues women were coping with. Younger women, those who had below average household income, women who had not completed year 12, women without a partner and women who smoked throughout pregnancy were more likely to attend a first antenatal check-up at ≥13 weeks' gestation, and to attend less than five visits (Table 3). Women who reported stressful life events or social health issues were also more likely to attend late, and to attend fewer visits.

**Table 3:** Risk factors by pattern of health service use

	Timing of first antenatal visit			Number of antenatal visits			
	**≤12 weeks'** **gestation ** **No. (%)**	**> 12 weeks'** **gestation ** **No. (%)**	**Odds Ratio** **(95% CI)**	**< 5 visits** **No. (%)**	**5-8 visits** **No. (%)**	**> 8 visits** **No. (%)**	**Odds Ratio** (95% CI)**

**Maternal age**							
≤24 years	365 (88.2)	49 (11.8)	**1.00 ref**	29 (7.3)	168 (42.4)	199 (50.3)	**1.00 ref**
≥25 years	3597 (95.2)	180 (4.8)	**2.68** **(1.9-3.7)**	144 (3.9)	1503 (40.1)	2048 (55.4)	**1.95** **(1.3-2.9)**

**Adjusted household income (AUD)**							
							
< $20,000	839 (89.7)	96 (10.3)	**2.94** **(2.1-4.0)**	53 (5.9)	412 (45.6)	439 (48.6)	**1.62** **(1.1-2.3)**
$20,000-$40,000	1878 (96.3)	73 (3.7)	**1.00 ref**	71 (3.7)	770 (40.3)	1072 (56.0)	**1.00 ref**
> $40,000	941 (96.2)	37 (3.8)	**1.01** **(0.7-1.5)**	30 (3.1)	372 (38.6)	561 (58.3)	**0.83** **(0.5-1.3)**

**Educational attainment**							
Completed year 12	3243 (94.9)	173 (5.1)	**1.00 ref**	118 (3.5)	1351 (40.4)	1875 (56.1)	**1.00 ref**
Did not complete year 12	821 (92.5)	67 (7.6)	**1.53** **(1.1-2.0)**	59 (6.9)	360 (41.9)	440 (51.2)	**2.02** **(1.5-2.8)**

**Maternal country of birth**							
Australia	3330 (94.8)	182 (5.2)	**1.00 ref**	129 (3.7)	1375 (40.0)	1936 (56.3)	**1.00 ref**
Overseas - English speaking	247 (91.8)	22 (8.2)	**1.63** **(1.0-2.6)**	16 (6.3)	117 (45.7)	123 (48.1)	**1.71** **(1.0-2.9)**
Overseas - Non-English speaking	484 (93.3)	35 (6.7)	**1.32** **(0.9-1.9)**	29 (5.8)	216 (43.1)	256 (51.1)	**1.58** **(1.0-2.4)**

**Relationship status**							
Married	3155 (95.7)	143 (4.3)	**1.00 ref**	118 (3.6)	1292 (40.0)	1821 (56.4)	**1.00 ref**
Living with partner	764 (92.0)	66 (8.0)	**1.9** **(1.4-2.6)**	44 (5.5)	349 (43.4)	412 (51.2)	**1.52** **(1.0-2.2)**
Single/divorced/separated	161 (84.3)	30 (15.7)	**4.11** **(2.7-6.3)**	16 (8.9)	76 (42.2)	88 (48.9)	**2.57** **(1.5-4.4)**

**Risk of complications in pregnancy**							
							
Lower risk	2470 (93.9)	160 (6.1)	**1.0 ref**	108 (4.2)	1147 (44.8)	1306 (51.0)	**1.00 ref**
Higher risk	1622 (95.2)	82 (4.8)	**0.78** **(0.6-1.0)**	71 (4.3)	576 (34.5)	1022 (61.2)	**1.01** **(0.7-1.4)**

**Maternal pre-pregnant BMI**							
Underweight (< 18.5)	167 (92.8)	13 (7.2)	**1.41** **(0.8-2.6)**	8 (4.6)	67 (38.3)	100 (57.1)	**1.05** **(0.5-2.2)**
Normal weight (18.5-24.9)	2268 (94.8)	125 (5.2)	**1.00 ref**	102 (4.4)	963 (41.3)	1266 (54.3)	**1.00 ref**
Overweight (25.0-29.9)	855 (94.6)	49 (5.4)	**1.04** **(0.7-1.5)**	40 (4.5)	363 (40.7)	490 (54.9)	**1.02** **(0.7-1.5)**
Obese (≥30)	546 (94.0)	35 (6.0)	**1.16** **(0.8-1.7)**	15 (2.6)	226 (39.7)	328 (57.6)	**0.59** **(0.3-1.0)**

**Smoking in pregnancy**							
Non-smoker	3310 (94.8)	181 (5.2)	**1.00 ref**	124 (3.6)	1377 (40.4)	1906 (55.9)	**1.00 ref**
Quit in pregnancy	327 (93.7)	22 (6.3)	**1.23** **(0.8-1.9)**	13 (3.8)	142 (41.3)	189 (54.9)	**1.04** **(0.6-1.9)**
Smoking throughout pregnancy	363 (91.2)	35 (8.8)	**1.76** **(1.2-2.6)**	34 (8.8)	166 (42.9)	187 (48.3)	**2.55** **(1.7-3.8)**

**Stressful life events/social health issues**							
None	1361 (95.3)	67 (4.7)	**1.00 ref**	40 (2.9)	551 (39.5)	805 (57.7)	**1.00 ref**
One to two issues	2009 (94.3)	122 (5.7)	**1.36** **(0.9-2.0)**	94 (4.5)	865 (41.4)	1130 (54.1)	**1.6** **(1.1-2.3)**
≥3 issues	709 (93.0)	53 (7.0)	**2.06** **(1.3-3.1)**	44 (6.0)	301 (41.1)	387 (52.9)	**2.17** **(1.4-3.4)**

** Odds ratios in the last column refer to comparisons of women attending <5 visits with women attending ≥ 5 visits.

### Associations with low birthweight

Univariable associations between maternal characteristics and low birthweight (< 2500) are shown in Table 4. Women at higher risk of obstetric complications in pregnancy had a fourfold increase in odds of having a low birthweight infant. Other maternal factors significantly associated with low birthweight were: not completing secondary education, being born overseas, and having a low pre-pregnant BMI (< 18.5). Women reporting three or more stressful life events or social health issues had a twofold increase in odds of having a baby with a low birthweight compared with women reporting no social health issues.

**Table 4:** Associations with infant birthweight

	≥2500 g No. (%)	< 2500g No. (%)	OR (95% CI)
**Maternal age**			
≤24 years	3467 (95.8)	151 (4.2)	**1.00 ref**
> 24 years	376 (95.4)	18 (4.6)	**1.10 (0.7-1.8)**

**Adjusted household income (AUD)**			
< $20,000	830 (95.7)	37 (4.3)	**1.00 (0.7-1.5)**
$20,000-$40,000	1799 (95.7)	81 (4.3)	**1.00 ref**
> $40,000	904 (95.9)	39 (4.1)	**1.00 (0.6-1.4)**

**Educational attainment**			
Completed year 12	3147 (96.1)	127 (3.9)	**1.00 ref**
Did not complete year 12	789 (94.0)	50 (6.0)	**1.57 (1.1-2.2)**

**Relationship status**			
Married	3035 (95.7)	135 (4.3)	**1.00 ref**
Living with partner	766 (96.0)	32 (4.0)	**0.93 (0.6-1.4)**
Single/divorced/separated	160 (93.0)	12 (7.0)	**1.69 (0.9-3.1)**

**Maternal Country of birth**			
Australia	3230 (96.1)	130 (3.9)	**1.00 ref**
Overseas - English speaking	241 (93.8)	16 (6.2)	**1.65 (1.0-2.8)**
Overseas - Non-English speaking	457 (93.5)	32 (6.5)	**1.73 (1.2-2.6)**

**Risk of complications in pregnancy**			
Lower risk	2473 (98.1)	49 (1.9)	**1.00 ref**
Higher risk	1500 (92.0)	130 (8.0)	**4.37 (3.1-6.1)**

**Maternal pre-pregnant BMI**			
Underweight (< 18.5)	163 (92.6)	13 (7.4)	**1.74 (1.0-3.2)**
Normal weight (18.5-24.9)	2193 (95.6)	100 (4.7)	**1.00 ref**
Overweight (25.0-29.9)	837 (96.0)	35 (4.0)	**0.92 (0.6-1.4)**
Obese (≥30)	537 (96.4)	20 (3.6)	**0.82 (0.5-1.3)**

**Smoking in pregnancy**			
Non-smoker	3222 (96.1)	132 (3.9)	**1. 00 ref**
Quit in pregnancy	325 (96.0)	13 (3.9)	**0.98 (0.5-1.7)**
Smoking throughout pregnancy	343 (92.4)	28 (7.6)	**1.99 (1.3-3.0)**

**Stressful life events/social health issues**			
None	1332 (96.8)	44 (3.2)	**1.00 ref**
One to two issues	1954 (95.7)	88 (4.3)	**1.36 (1.0-2.0)**
≥3 issues	676 (93.6)	46 (6.4)	**2.06 (1.3-3.1)**

In order to obtain a more precise estimate of the association between low birthweight and the number of stressful life events and social health issues, we developed the four models shown in Table 5. Maternal smoking status was fitted in all models for *a priori *reasons based on the known aetiological relationship between maternal smoking and infant birthweight. The first model adjusts for maternal smoking alone, and shows that the association between number of stressful life events and social health issues and low birthweight remains statistically significant after adjusting for maternal smoking in pregnancy. Model 2 adjusts for other socio-demographic factors associated with the exposure of main interest (three or more social health issues) and the outcome variable (low birthweight) in univariable analyses. Models 3 and 4 include smoking status and all of the socio-demographic variables included in model 2, and adjust separately for number of antenatal visits (model 3) and risk of complications in pregnancy (model 4). All models indicate a dose effect related to number of stressful life events and social health issues. All models that fitted maternal country of birth indicated that women born overseas in non-English speaking countries had significantly raised odds of having a low birthweight infant.

**Table 5:** Multivariable logistic regression analyses assessing associations with low birthweight (n = 3650)

	< 2500 grams No. (%)	Unadjusted OR (95% CI)	Model 1 Adj OR (95% CI)	Model 2 Adj OR (95% CI)s	Model 3 Adj OR (95% CI)	Model 4 Adj OR (95% CI)
**Stressful life events/social health issues**						
No issues	40 (3.3)	1.00 ref	**1.00 ref**	**1.00 ref**	**1.00 ref**	**1.00 ref**
1-2 issues	75 (4.1)	1.27 (0.9-1.9)	**1.26 (0.9-1.9)**	**1.24 (0.8-1.8)**	**1.22 (0.8-1.8)**	**1.13 (0.8-1.7)**
≥3 issues	38 (6.2)	1.94 (1.2-3.1)	**1.80 (1.1-2.9)**	**1.77 (1.1-2.8)**	**1.72 (1.1-2.8)**	**1.44 (1.0-2.3)**

**Smoking status**						
Non-smoker	120 (4.0)	1.00 ref	**1.00 ref**	**1.00 ref**	**1.00 ref**	**1.00 ref**
Quit in pregnancy	9 (3.0)	0.76 (0.4-1.5)	**0.72 (0.36-1.4)**	**0.69 (0.3-1.4)**	**0.71 (0.4-1.4)**	**0.67 (0.3-1.3)**
Smoking throughout pregnancy	24 (7.5)	1.97 (1.3-3.1)	**1.76 (1.1-2.8)**	**1.75 (1.1-2.8)**	**1.57 (1.0-2.6)**	**1.82 (1.1-3.0)**

**Maternal country of birth**						
Australia	113 (3.8)	1.00 ref		**1.00 ref**	**1.00 ref**	**1.00 ref**
Overseas - English speaking	13 (5.8)	1.58 (0.9-2.8)		**1.66 (0.9-3.0)**	**1.54 (0.8-2.8)**	**1.60 (0.9-2.9)**
Overseas - Non-English speaking	27 (6.4)	1.76 (1.1-2.7)		**1.85 (1.2-2.9)**	**1.80 (1.1-2.8)**	**1.90 (1.2-3.0)**

**Maternal pre-pregnant BMI**						
Underweight (< 18.5)	11 (6.8)	1.62 (0.8-3.1)		**1.37 (0.7-2.6)**	**1.37 (0.7-2.6)**	**1.39 (0.7-2.7)**
Normal weight (18.5-24.9)	93 (4.3)	1.00 ref		**1.00 ref**	**1.00 ref**	**1.00 ref**
Overweight (25-29.9)	33 (4.0)	0.92 (0.6-1.4)		**0.92 (0.6-1.4)**	**0.94 (0.6-1.4)**	**0.81 (0.5-1.2)**
Obese (≥30)	16 (3.1)	0.69 (0.4-1.2)		**0.69 (0.4-1.2)**	**0.72 (0.4-1.2)**	**0.54 (0.3-0.9)**

**Educational attainment**						
Completed year 12	113 (3.9)	1.00 ref		**1.00 ref**	**1.00 ref**	**1.0 ref**
Did not complete year 12	40 (5.4)	1.46 (1.0-2.1)		**1.36 (0.9-2.0)**	**1.30 (0.9-1.9)**	**1.27 (0.9-1.9)**

**Number of antenatal visits**						
≥5 visits	133 (3.8)	1.00 ref			**1.00 ref**	
< 5 visits	20 (14.3)	4.23 (2.6-7.0)			**3.48 (2.1-5.8)**	

**Risk of complications in pregnancy**						
Lower risk	40 (1.8)	1.00 ref				**1.00 ref**
Higher risk	113 (7.9)	4.72 (3.3-6.8)				**4.83 (3.3-7.0)**

**Model 1: **exposures of main interest adjusted for smoking status

**Model 2**: exposure of main interest adjusted for smoking status, maternal country of birth, maternal BMI, maternal education.

**Model 3: **exposure of main interest adjusted for smoking status, maternal country of birth, maternal BMI, maternal education and number of antenatal visits

**Model 4: **exposure of main interest adjusted for smoking status, maternal country of birth, maternal BMI, maternal education and risk of complications in pregnancy

## Discussion

Over 30 years ago Archie Cochrane noted that antenatal care had escaped critical assessment in terms of content [33]. There is still a paucity of research evaluating the effectiveness of antenatal interventions specifically targeting modifiable risk factors for poor maternal and/or child health outcomes [34-36]. Recent efforts to improve the evidence base for antenatal care have focused on clinical care, smoking cessation support and on assessment of recommended visit schedules [37-40]. The relationship between the *content *and *organisation *of antenatal care and subsequent maternal and child health outcomes has received scant attention [36]. For example, scientific knowledge about the extent to which different *approaches to care *(e.g. model of maternity care, pregnancy outreach workers) may influence nutrition, exercise habits, oral health, smoking, drug use, choices regarding infant feeding method, maternal depression or anxiety, intimate partner violence, inter pregnancy interval, and post pregnancy use of health services is negligible.

Our findings from a large Australian population-based survey of recent mothers attest to the high prevalence of potentially modifiable risk factors such as smoking and overweight and obesity. They also highlight a concerning level of social adversity associated with stressful life events and social health issues co-occurring with pregnancy. One in six women reported three or more stressful life events or social health issues in the 12 months before the birth. These women were much more likely to perceive that they were discriminated against in health care settings, to attend later in pregnancy and to have fewer antenatal visits. Women coping with multiple life events or social health issues remain significantly more likely to have a low birthweight infant when maternal smoking, number of antenatal visits, and other covariates are taken into account. Interpretation of the relationship between timing and number of antenatal visits and infant birthweight is complex. Our data show that some groups of women are less likely to access antenatal care early, and to attend regularly during pregnancy. The same groups of women are more likely to have adverse outcomes. This does not signify causation, but it does suggest that women who most need care and support during pregnancy are more likely to not to engage with services.

Several study limitations need to be considered. First, we used a modified life events scale originally developed for use in pregnancy [41] that has also been used in large scale maternity surveys in the United States and Canada [16,30,42]. Modifications made based on piloting and consultations preceding implementation of the HMHF Survey have not been subject to psychometric testing. Nor have we sought in this paper to differentiate different types of stressors. Our primary aim was to assess the association between low birthweight and number of stressful life events and social health issues and consider potential implications for the care of women during pregnancy. Consideration of broader questions regarding the relationship between different types of stress in pregnancy and area level deprivation are beyond the scope of analyses presented in the paper.

Second, it is plausible that women having a low birthweight infant may be more inclined to recall adverse life events in pregnancy than women who have an infant of higher birthweight, although this potential source of bias may have been lessened by the time elapsed between the birth and completing the survey at around six months postpartum. Third, we were not able to validate maternal recall of infant birthweight or maternal smoking in pregnancy. Validation studies comparing mothers' accounts of infant birthweight with medical records have shown high levels of agreement for this outcome as opposed to gestation, where there are more mixed findings [43,44]. This was the reason for reporting infant birthweight, rather than small for gestational age. We acknowledge the potential for misclassification of maternal smoking status in early and late pregnancy. However, our finding that 17.6% of study participants reported smoking in pregnancy is in accord with routinely collected data available for seven Australian states and territories in 2007 which indicate that 16.6% of women smoked in pregnancy [25]. We used a multiple response format for seeking information about maternal smoking which has been shown to improve disclosure of maternal smoking in self-administered questionnaires [29]. Fourth, the sample while relatively large, provided low numbers in sub-groups for some comparisons (e.g. single women, Indigenous women).

Strengths of the study include a population-based sample drawn from two out of the eight Australian states and territories. The sampled population for the survey comprised 15% of confinements in South Australia and 8% in Victoria in 2007. While it is not possible to determine causal pathways in an observational study, the findings draw attention to social circumstances in women's lives that may place them at higher risk of poor perinatal and longer term outcomes. They also draw attention to vulnerable population groups who may be at additional risk.

## Conclusions

Developing the infrastructure to support an integrated primary care approach to antenatal care in which services have the capacity to respond effectively to social circumstances in women's lives and to vulnerable populations is long overdue, and requires far reaching changes involving both hospital and community based services [45]. The recently released NICE clinical guideline on service provision for pregnant women with complex social factors provides an excellent starting point and practical recommendations focusing on four vulnerable population groups: pregnant women who misuse substances, young pregnant women, pregnant women experiencing intimate partner violence and pregnant women who are recent migrants, asylum seekers or refugees [46]. Another important area for intervention is pre-conception, especially during adolescence and during early adulthood. There are major challenges for policy makers and health services in developing an appropriately skilled workforce; development of a life-course approach to promotion of maternal, newborn and child health that includes a focus on public health intervention during pregnancy and prior to conception; development of team work and collaboration within and across agencies; facilitating professional, consumer and community participation; and investment in monitoring and evaluation to ensure outcome based accountability. A shift in this direction requires fundamentally different thinking and co-ordinated action across sectors to bring about system wide changes [47,48]. Only if this challenge is embraced will health care services be capable of developing effective proactive approaches to potentially modifiable risks and health needs in diverse populations.

## Competing interests

The authors declare that they have no competing interests.

## Authors' contributions

SB and JY conceived the study; PB and JR contributed to the study protocol and grant applications for the study; JY and PB supervised data collection; JY and GW are responsible for data management; GS undertook analyses reported in the paper; SB wrote the manuscript. All authors contributed to interpretation of data and commented on drafts of the paper. SB is guarantor.

## Pre-publication history

The pre-publication history for this paper can be accessed here:

http://www.biomedcentral.com/1471-2458/11/196/prepub

## Supplementary Material

Additional file 1 **Measures of stressful life events and social health issues and perceived discrimination**. A complete list of the items used in this survey to measure stressful life events and social health issues and the five questions adapted from the Measure of Indigenous Racism Experience to elicit information about women's experience of discrimination by health professionals in the perinatal period. Click here for file
