# Supplementary material for: Stressful life events, social health issues and low birthweight in an Australian population-based birth cohort: challenges and opportunities in antenatal care
Source: BMC Public Health. 2011 Mar 30;11:196. doi: 10.1186/1471-2458-11-196 (PMC3080815; doi:10.1186/1471-2458-11-196)
Supplement: Additional file 1 — Measures of stressful life events and social health issues and perceived discrimination. A complete list of the items used in this survey to measure stressful life events and social health issues and the five questions adapted from the Measure of Indigenous Racism Experience to elicit information about women's experience of discrimination by health professionals in the perinatal period. [file 1471-2458-11-196-S1.DOC]

**Additional file 1**

**1. Measure of stressful life events and social health issues in the 12 months before the index birth**

**Did any of the following things happen to you in the 12 months prior before your new baby was born?**

For each item, tick yes if it happened to you, and no if it did not.

You had a major illness or injury

A close family member or friend had a major illness or injury

You started a new close personal relationship

You got married or moved in with your partner

Separation or divorce

You moved to a new house/new place to live

You were homeless

You lost your job when you wanted to go on working

Your partner lost his/her job

Your partner said he/she did not want you to be pregnant

You were humiliated or emotionally abused in other ways by your partner or ex partner

You had a lot of bills you couldn’t pay

You didn’t have enough money to buy food

You had a miscarriage

You had treatment for infertility

There was serious conflict between members of your family

Death of a close family member or friend

You were kicked, hit, slapped or otherwise physically hurt by your partner or ex partner

Someone else (other than your partner or ex partner) pushed, grabbed, shoved, kicked or hit you

You or your partner had trouble with alcohol or illicit drugs

Your or your partner had trouble with gambling

You were forced to take part in unwanted sex

You or your partner had legal troubles or were involved in a court case

NONE OF THESE

**2. Measure of perceived discrimination in health care settings**

The following questions ask about experiences of being treated unfairly, or discriminated against by health professionals. Being ‘treated unfairly’ means being treated as if you were inferior, rudely, with disrespect, being ignored, insulted, stereotyped, harassed or having unfair assumptions made about you.

**Over the past year, have any of the following things happened to you?**

Doctors, midwives, nurses or other health professionals treated you with less courtesy and respect than other people

You received poorer care from doctors, midwives, nurses or other health professionals than other people

Doctors, midwives, nurses or other health professionals talked down to you

Doctors, midwives, nurses or other health professionals treated you with less respect than other people

You were insulted, stereotyped or ignored by doctors, midwives, nurses or other health professionals
